# Mental and behavioral abnormalities caused by occlusion of the right coronary artery: A case report

**DOI:** 10.1097/MD.0000000000044315

**Published:** 2025-09-05

**Authors:** Weixian Xu, Miaozhen Chen, Yunhai Zhang, Zhimin Luo

**Affiliations:** a Department of Intensive Care Unit, The Eighth Clinical Medical College of Guangzhou University of Chinese Medicine, Foshan Hospital of Traditional Chinese Medicine, Foshan City, Guangdong Province, China; b Department of Integrated Traditional Chinese and Western Medicine, Foshan Chancheng District People’s Hospital Nanzhuang Hospital, Foshan City, Guangdong Province, China.

**Keywords:** abnormal behavior, arrhythmia, case report, Inferior wall myocardial infarction, sinus arrest

## Abstract

**Rationale:**

Mental and behavioral abnormalities are difficult neurological conditions, and the site of the lesion may involve the basal ganglia. Its etiology is varied and requires a detailed differential diagnosis.

**Patient concerns:**

An 81-year-old woman had a history of “cerebral infarction” for 5 years. She could walk independently and take care of herself without the assistance of others. This time the chief complaint is generalized fatigue for 4 days and mental and behavioral abnormalities for 1 day.

**Diagnoses:**

the right coronary artery occlusion.

**Interventions:**

We implanted a drug-eluting stent, then helped her with mechanical ventilation, and implanted a temporary pacemaker.

**Outcomes:**

She recovered and can walk alone with a walker.

**Lessons:**

Right coronary artery infarction often leads to a hypotensive state, shock, and arrhythmias such as bradycardia. Transient ischemic attack or cerebral infarction may be induced if the patient has neck or cerebrovascular lesions. Cranial computed tomography should be performed rapidly in patients presenting with mental and behavioral abnormalities. Simultaneous electrocardiogram (ECG), cardiac ultrasound and myocardial injury marker tests should also be conducted to ensure timely and accurate diagnosis.

## 1. Introduction

Mental and behavioral abnormalities are difficult neurological conditions. Its etiology is varied and requires a detailed differential diagnosis. Cardiac lesions that cause hemodynamic derangements and fail to provide adequate perfusion to the brain will likely induce neurological disorders.

## 2. Case

### 2.1. Patient concerns

An 81-year-old woman presented to Foshan Hospital of Traditional Chinese Medicine on December 28, 2023 with “generalized fatigue for 4 days and mental and behavioral abnormalities for 1 day.” She had a history of “cerebral infarction” for 5 years. She could walk independently and take care of herself without the assistance of others. The patient’s family said she developed generalized weakness with vomiting 4 days ago, and mental and behavioral abnormalities for 1 day. The patient was admitted in a wheelchair with a painful face, generalized weakness, and inability to communicate. She was unable to control herself at times by shouting and removing clothing.

Body checking cannot be performed cooperatively. Temperature: 36.4°C, Pulse: 50 beats/minute, Respiratory rate: 20 beats/minute, Blood Pressure: 132/62 mmHg. Her words were confusing. Muscle tension is normal. Grading Muscle Strength is IV. Her Babinski sign and Brudzinski sign were negative.

**Figure 1.**
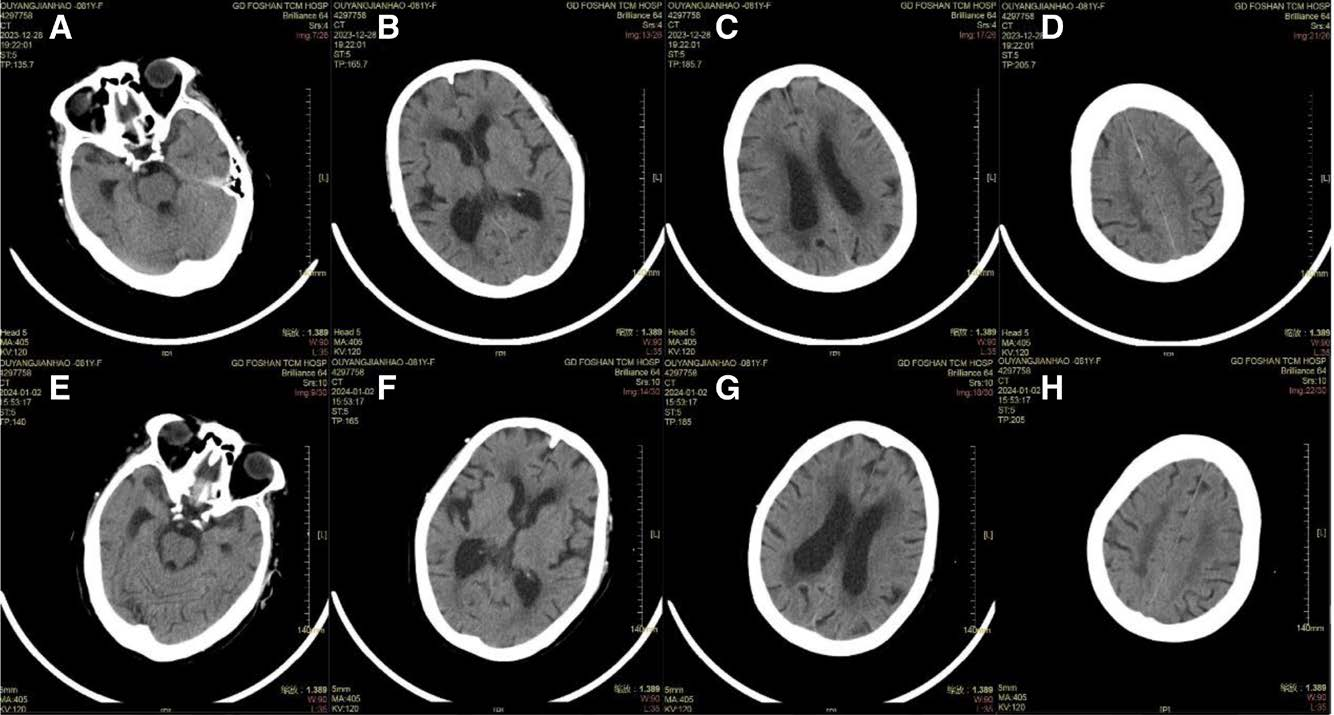
Patient’s CT. (A–D) shows cranial CT on December 28, 2023, suggesting cerebral atrophy and cerebral white matter sparing. (E and F) shows cranial CT on January 2, 2024, suggesting focal cerebral infarction in the right thalamus and right occipital lobe. CT = computed tomography.

Admitted to the Neurology Department, we performed a cranial computed tomography (CT) for her, suggesting cerebral atrophy and cerebral white matter sparing (see Fig. 1A–D), and gave her treatment for secondary prevention of cerebrovascular disease.

## 3. Diagnosis

**Figure 2.**
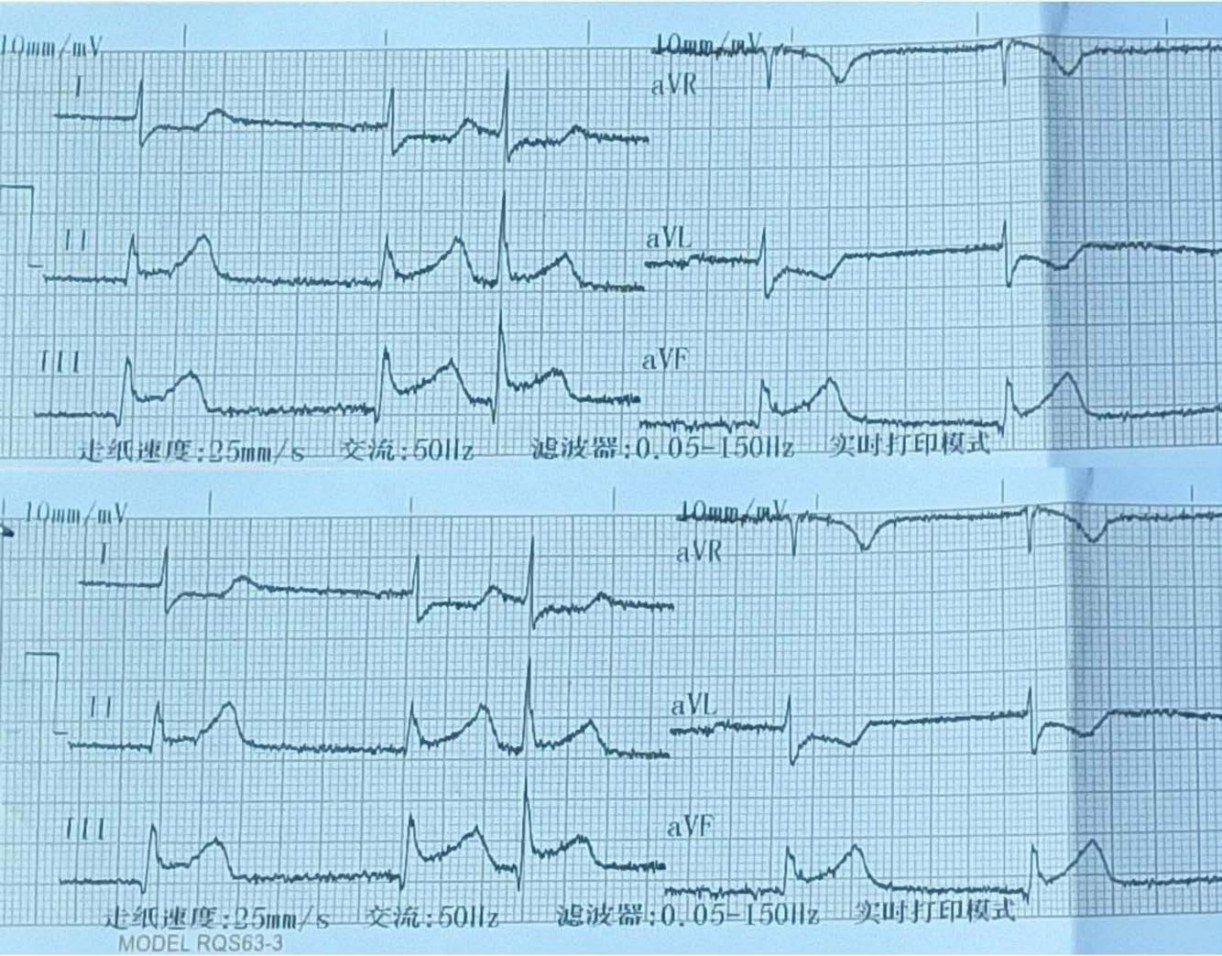
ECG at 10 pm on December 28, 2023, suggested acute inferior wall myocardial infarction. ECG = electrocardiogram.

**Figure 3.**
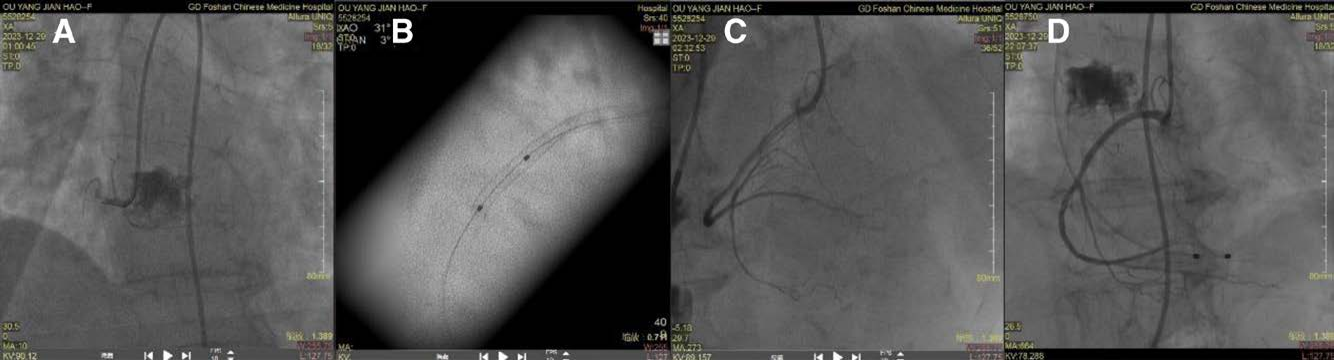
Coronary angiography. (A) shows occlusion of the RCA. (B) shows implantation of a stent. (C) shows the RCA after stent implantation. (D) shows the RCA after implantation of a temporary pacemaker. RCA = right coronary artery.

**Figure 4.**
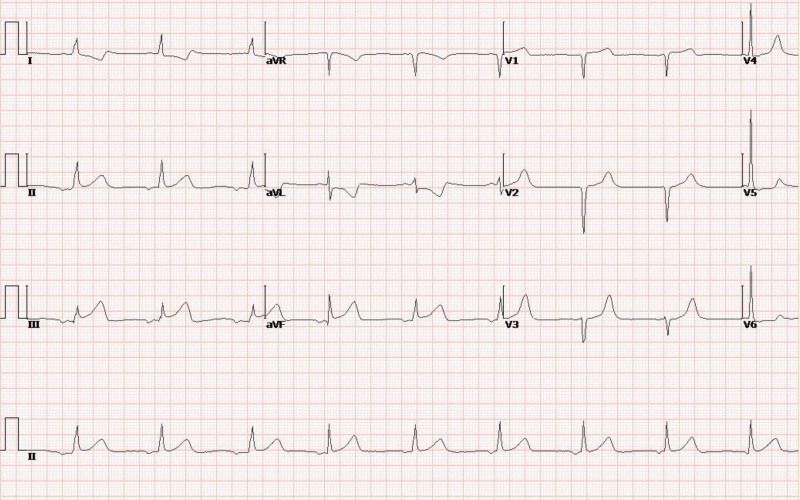
ECG at 8 am on December 29, 2023, suggested atrial and atrioventricular junctional escape rhythms. ECG = electrocardiogram.

On that night, her Troponin I was 39.496 µg/L. We immediately performed an ECG, which suggested acute inferior wall myocardial infarction (see Fig. 2), so we referred her to the cardiology department for emergency coronary intervention. Coronary angiography (CAG) found complete occlusion of the right coronary artery (RCA) opening beyond, with a flow the thrombolysis in myocardial infarction risk score of 0. Then we implanted a drug-eluting stent (see Fig. 3A–C). The next morning, her ECG suggested atrial and atrioventricular junctional escape rhythms (see Fig. 4). The patient still yelled and taken off the clothes unconsciously, accompanied by low blood pressure and a slow heart rate, requiring dopamine and mesalamine.

## 4. Interventions

The second night, the cardiac monitor indicated that she was in cardiac arrest, and we immediately performed cardiopulmonary resuscitation. After 30s of chest compressions, she regained voluntary rhythm. We helped her with mechanical ventilation, implanted a temporary pacemaker (see Fig. 3D), and transferred her to the ICU for continued treatment.

**Figure 5.**
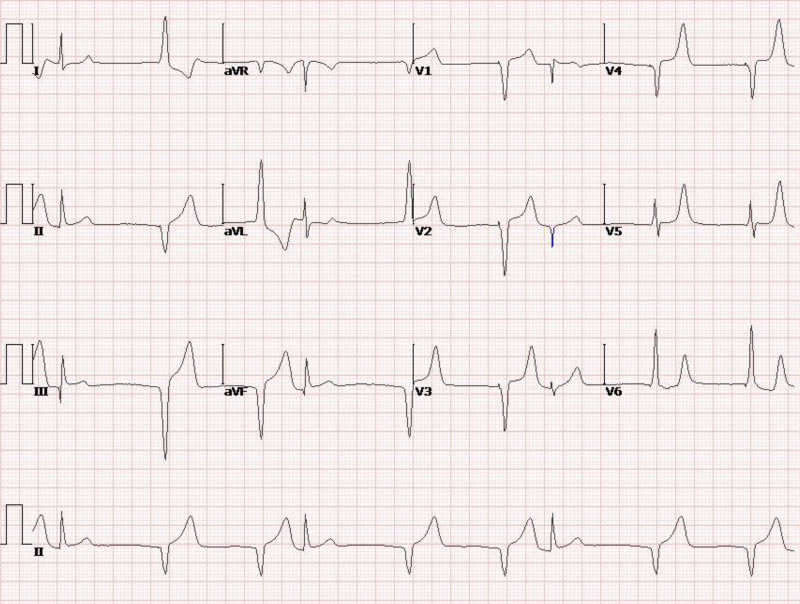
ECG at 8 am on December 30, 2023, shows pacemaker rhythm and anterior interventricular Q wave. ECG = electrocardiogram.

**Figure 6.**
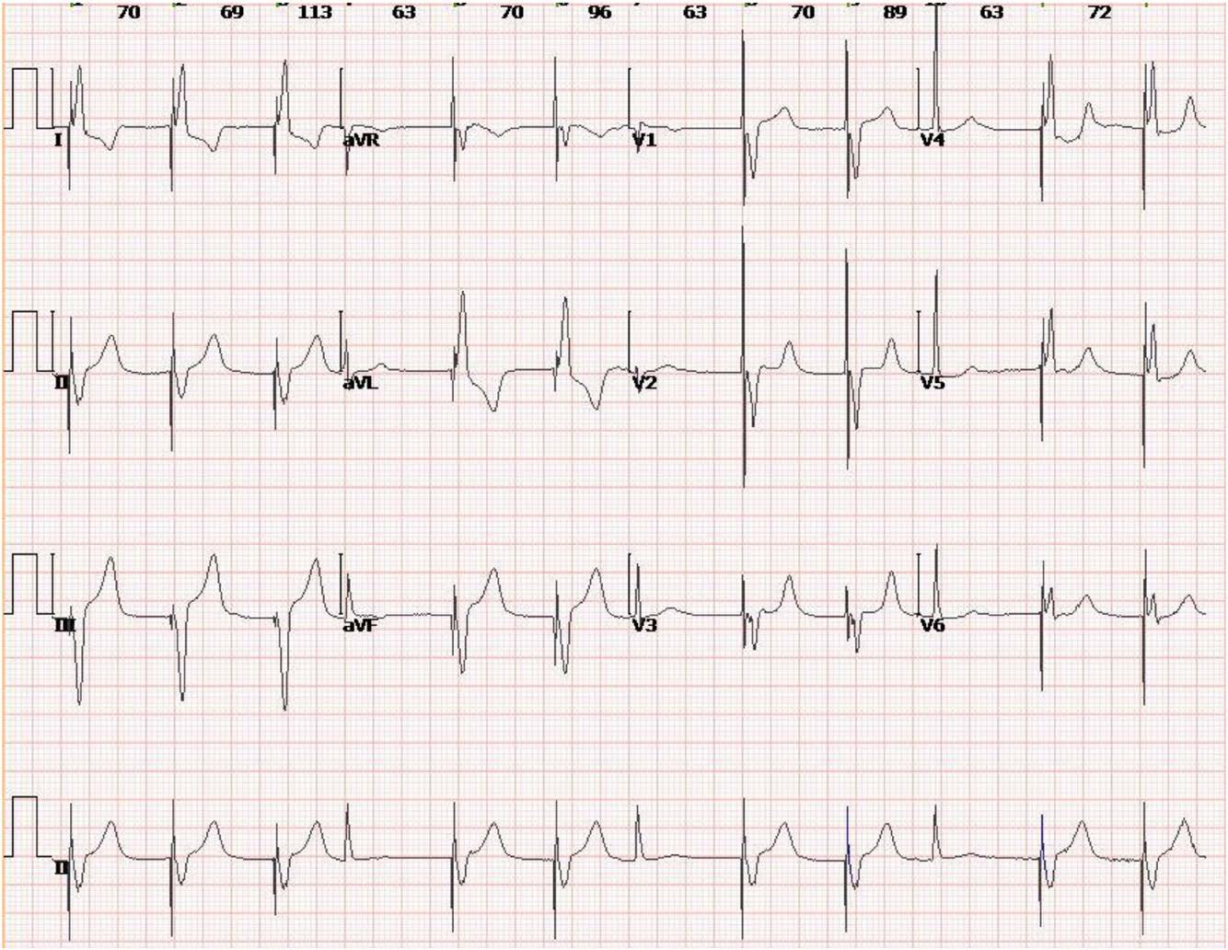
ECG at 11 am on December 31, 2023, shows pacemaker rhythm. ECG = electrocardiogram.

**Figure 7.**
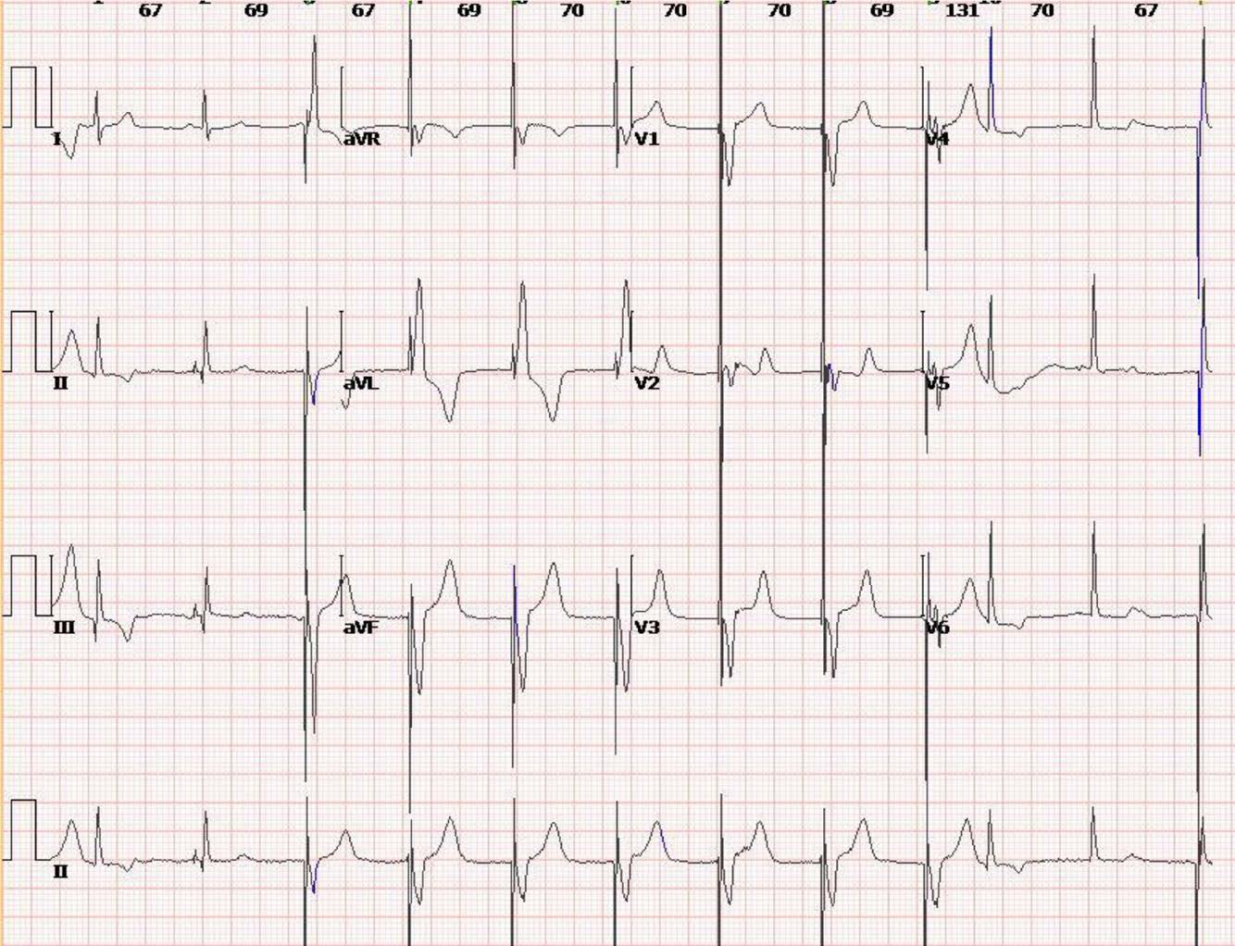
ECG at 4 pm on December 31, 2023, shows pacemaker rhythm and accelerated atrial premature beats. ECG = electrocardiogram.

**Figure 8.**
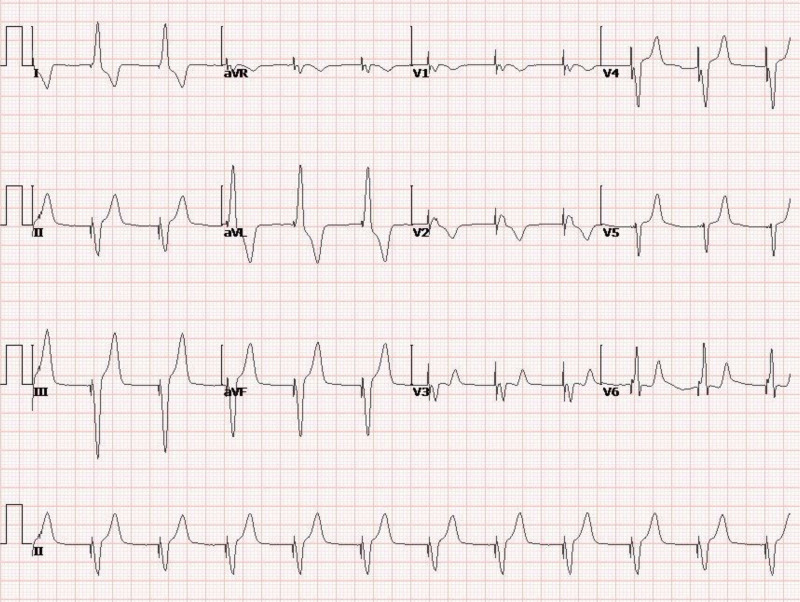
ECG at 8 am on January 1, 2024, shows pacemaker rhythm. The patient did not experience AV block or arrest. ECG = electrocardiogram.

The patient’s blood pressure and heart rate gradually stabilized, troponin I and myoglobin decreased gradually (see Table 1), and atrioventricular block(AVB) gradually recovered (see Figs. 5–7). On day 5, we adjusted the pacemaker trigger threshold and the patient did not experience AVB or arrest (see Fig. 8). Repeat CT of the cranium suggested focal cerebral infarction in the right thalamus and right occipital lobe. (see Fig. 1E–H).

**Table 1:** Changes in myoglobin and troponin I.

	Day 1	Day 2	Day 3	Day 4	Day 5	Day 6	Day 7
Myoglobi (ng/L)	–	903.27	824.27	283.87	266.71	137.23	164.23
Troponin I (µg/L)	39.496	68.759	41.966	19.341	9.475	8.027	5.163

The changes in the patient’s Myoglobin and Troponin I were consistent with acute myocardial infarction, which decreased gradually.

**Figure 9.**
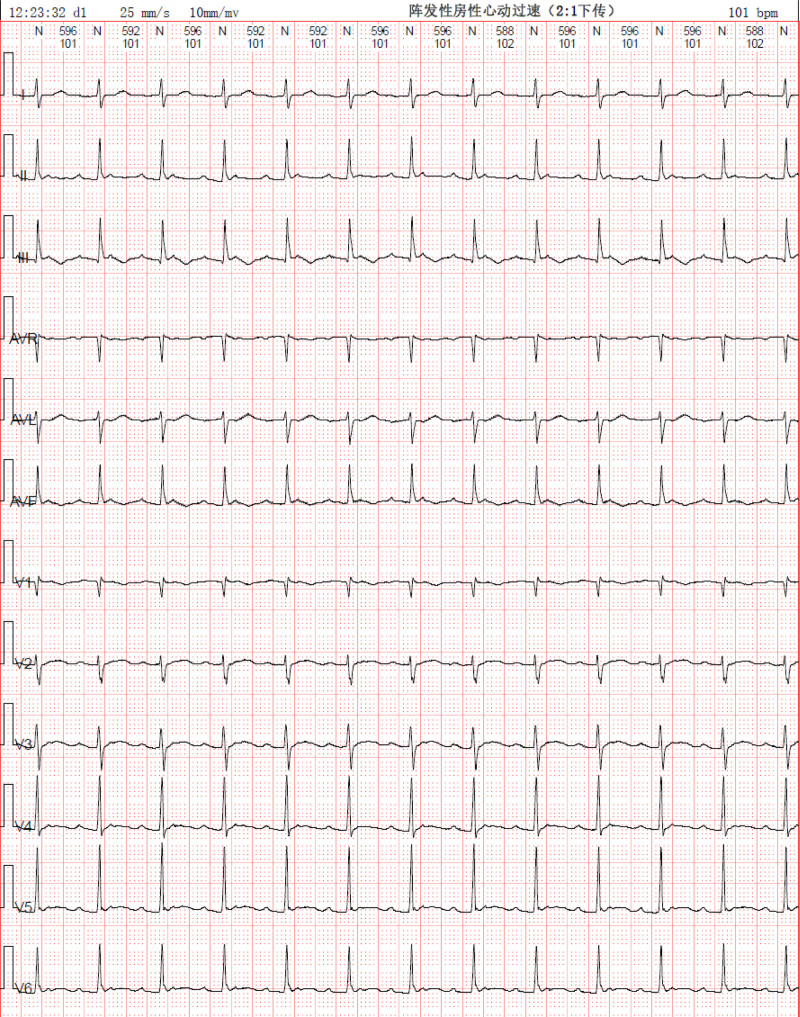
Ambulatory electrocardiogram on January 3, 2024, showed paroxysmal atrial tachycardia with occasional multiple premature ventricular beats.

On day 7, the patient’s ambulatory electrocardiogram showed paroxysmal atrial tachycardia with occasional multiple premature ventricular beats (see Fig. 9), so the temporary pacemaker was withdrawn and the patient was transferred to a community hospital for further treatment.

**Figure 10.**
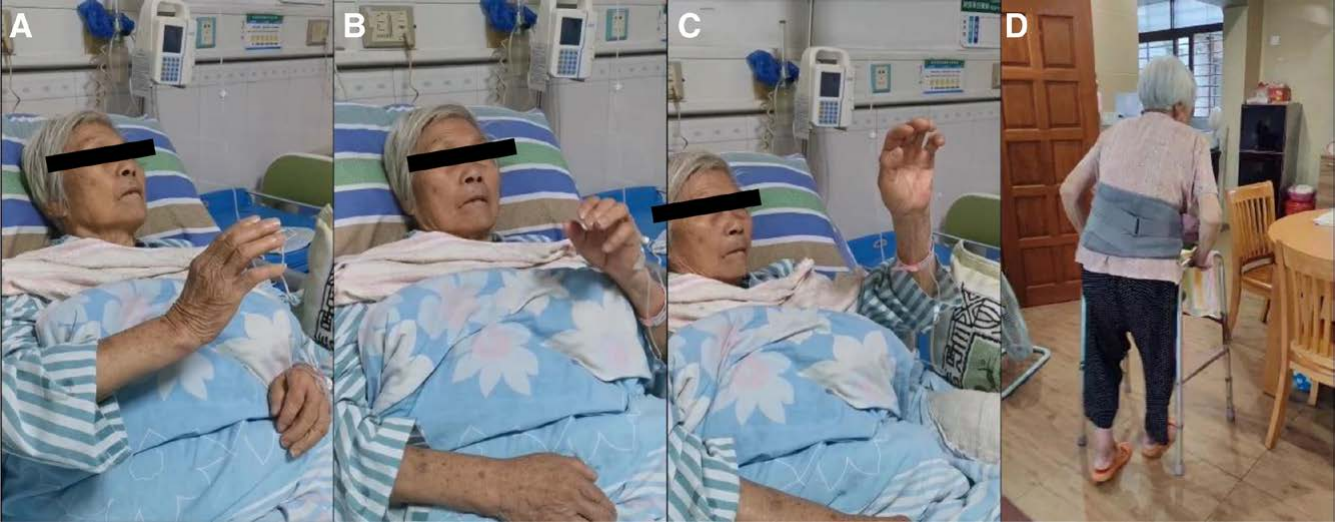
Clinical character. (A–C) shows the patient is apathetic and grasps objects aimlessly into the air with both hands. (D) shows she could walk alone with a walker.

After day 8, the patient was awake and evacuated from the ventilator and tracheal tube. The patient is apathetic and grasps objects aimlessly into the air with both hands (see Fig. 10A–C). After 1 month of rehabilitation, the patient recovered mentally and discontinued his abnormal behavior.

### 4.1. Outcomes

After 6 months of follow-up, she is now walking alone with a walker and taking care of herself without the assistance of others (see Fig. 10D).

## 5. Discussion

The patient came to the clinic with mental and behavioral abnormalities. On admission, she was shouting and involuntarily removing her clothes; during the recovery period, she was aimlessly grabbing objects in the air with both hands. This clinical presentation is similar to chorea in movement disorders, or tardive dyskinesia. Movement disorders are a group of neurological disorders mainly manifested by movement symptoms such as delayed random movements, involuntary movements, muscle tension abnormalities, and postural and gait disorders, and most of them are related to basal ganglia lesions.^[1,2]^ Its common causes are vascular diseases, infectious diseases, degenerative diseases, trauma, neoplasms, demyelinating diseases, metabolic and nutritional disorders, poisoning, hereditary diseases, and congenital malformations. Family history is important in patients with a chronic onset of the disease, as it can differentiate between hereditary and nonhereditary forms of chorea.^[3]^ However, in patients with an acute onset of disease, we first need to rule out cerebrovascular disease, such as infarction or hemorrhage.^[4]^

This patient had an onset of 4 days, and no obvious infarction or hemorrhage was seen on CT. After that, we continued to investigate related causes, and in the process of routine cardiovascular disease investigation, we inadvertently found that the patient’s troponin was elevated, and the electrocardiogram suggested that the ST-segment elevation in leads II, III, and aVF, which was considered to be an acute lower-wall myocardial infarction, and the coronary angiography suggested that the RCA opening was occluded.

The patient’s 2019 carotid vascular ultrasound at our institution suggested bilateral carotid intima-media thickening with multiple plaques, no abnormalities in the bilateral vertebral arteries left subclavian artery, and plaques in the beginning segment of the right subclavian artery, and no stenosis in the official lumen. We speculate that on the basis of carotid or intracranial arterial stenosis caused by various reasons, when there is hypotension or blood pressure fluctuation, the blood flow in the distal vessels of the stenosis site decreases, and cerebral ischemic symptoms can occur,^[5]^ and when the blood pressure is rebounded, the local cerebral blood flow returns to normal, and the symptoms of ischemia disappear.

The RCA is generally distributed in the right atrium, most of the anterior wall of the right ventricle, all of the lateral and posterior walls of the right ventricle, a portion of the posterior wall of the left ventricle and the posterior third of the interventricular septum, including the posterior half of the left bundle branch, as well as the atrioventricular node and sinoatrial node. Acute inferior wall myocardial infarction following RCA occlusion may result in a hypotensive state, shock, and bradyarrhythmias such as third-degree atrioventricular block, sinus bradycardia, or even sinus arrest.^[6]^

A drop in blood pressure is a common complication of AMI, when left uncorrected it can progress to cardiogenic shock, which is a cause of death.^[7]^ In the case of RCA occlusion, physicians are overly concerned that infusion will induce or exacerbate pump failure, which results in too little rehydration and not enough to correct the preexisting hypovolemia.

Thrombolysis or emergency coronary intervention can restore reperfusion to ischemic myocardium in a timely and effective manner, but when reperfusion occurs, a large number of calcium ions enter the cells, leading to intracellular calcium overload and triggering arrhythmias.^[8]^ Hypotension is also common after early successful reperfusion in patients with inferior wall AMI because of the marked increase in vagal tone triggered by sympathetic overactivation.

Preinfarction angina is an independent protective factor against reperfusion injury. Patients with preinfarction angina are less prone to reperfusion arrhythmias, which may be an effect of ischemic preadaptation.^[9–11]^ In contrast, this patient presented with mental and behavioral abnormalities was unable to clarify his angina, had no preadaptation to ischemia, and was more prone to arrhythmias.

The site of AVB in inferior wall AMI is mostly in the AV node area or above the Hirschsprung bundle, so inferior wall AMI is prone to complicate AVB, but it rarely results in atrioventricular node necrosis, and most of them have a good prognosis.^[12]^ When the necessary heart rate cannot be maintained with medications, Rapid and accurate administration of pacing therapy in the shortest possible time should be sought.^[13]^

## 6. Conclusion

RCA infarction often leads to a hypotensive state, shock, and arrhythmias such as bradycardia. Transient ischemic attack or cerebral infarction may be induced if the patient has neck or cerebrovascular lesions. Cranial CT should be performed rapidly in patients presenting with mental and behavioral abnormalities. Simultaneous ECG, cardiac ultrasound and myocardial injury marker tests should also be conducted to ensure timely and accurate diagnosis.

## Author contributions

**Data curation:** Weixian Xu, Zhimin Luo.

**Formal analysis:** Zhimin Luo.

**Funding acquisition:** Weixian Xu, Zhimin Luo.

**Methodology:** Yunhai Zhang.

**Writing – original draft:** Miaozhen Chen.

**Writing – review & editing:** Weixian Xu.
